# Antifouling Zwitterionic Polymer Coatings for Blood-Bearing Medical Devices

**DOI:** 10.1021/acs.langmuir.4c04532

**Published:** 2025-01-27

**Authors:** Kagya Amoako, Rei Ukita, Keith E. Cook

**Affiliations:** † Department of Chemistry and Chemical and Biomedical Engineering, University of New Haven, West Haven, Connecticut 06516, United States; ‡ Department of Cardiac Surgery, Vanderbilt University Medical Center, Nashville, Tennessee 37232, United States; § Department of Biomedical Engineering, Vanderbilt University, Nashville, Tennessee 37240, United States; ∥ Department of Biomedical Engineering, Carnegie Mellon University, Pittsburgh, Pennsylvania 15213, United States

## Abstract

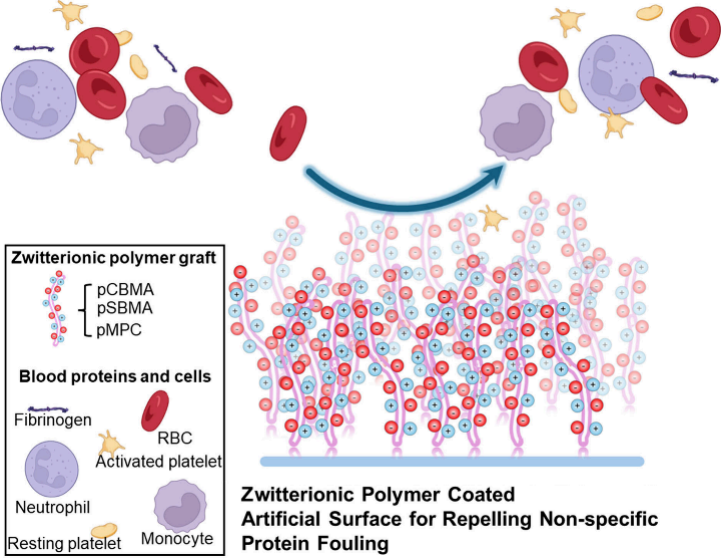

Blood-bearing medical devices are essential for the delivery of critical care medicine and are often required to function for weeks to months. However, thrombus formation on their surfaces can lead to reduced device function and failure and expose patients to systemic thrombosis risks. While clinical anticoagulants reduce device related thrombosis, they also increase patient bleeding risk. The root cause of device thrombosis and inflammation is protein adsorption on the biomaterial surfaces of these devices. Protein adsorption activates the coagulation cascade and complement, and this, in turn, activates platelets and white blood cells. Surface modifications with zwitterionic polymers are particularly effective at reducing protein adsorption as well as conformational changes in proteins due to their hydrophilicity. Multiple coating strategies have been developed using carboxybetaine (CB), sulfobetaine (SB), and 2-methacryloyloxyethyl phosphorylcholine (MPC) zwitterionic polymers applied to the metals and hydrophobic polymers that make up the bulk of blood-bearing medical devices. These coatings have been highly successful at creating large reductions in protein adsorption and platelet adhesion during studies on the order of hours on flat surfaces and at reducing thrombus formation for up to a few days in full medical devices. Future work needs to focus on their ability to limit inflammation, particularly during hemodialysis, and in providing anticoagulation on the order of weeks, particularly in artificial lungs.

## Introduction

Blood coagulation has, historically, been the most significant challenge in creating safe and effective blood-bearing medical devices. The development and use of vascular grafts and artificial heart valves, kidneys, hearts, and lungs have all been plagued by pathogenic blood clot formation.^1,2^ Coagulation occludes blood passages through the device, increasing blood flow resistance, covering key components, and ultimately, damaging the key functions of the medical device. Vascular grafts occlude and reduce tissue perfusion; dialyzers and artificial lungs lose their mass transfer function; and blood pumps shoot thromboemboli into the vasculature, causing downstream, native organ dysfunction or failure.

Historically, most of the solutions that have been employed to overcome clot formation in these devices have been only slightly better than the problem itself. The traditional method of combating clot formation is to deliver intravenous heparin to provide systemic anticoagulation. Heparin is inexpensive and effective at inhibiting the common coagulation cascade by increasing antithrombin III’s (ATII) inhibition of Factor Xa (FXa) and thrombin. Unfortunately, by inhibiting the common pathway of the coagulation cascade, it equally inhibits clot formation caused by artificial surfaces, driven by the intrinsic branch of the coagulation cascade, and clot formation in patient tissues, driven by the extrinsic branch. As a result, heparin creates a marked increase in bleeding risk that can create a similar, life-threatening risk as the coagulation itself.^3,4^ Numerous other systemic anticoagulants have been developed,^5−13^ and while there is hope for selective intrinsic branch anticoagulants,^6,7,12−14^ there is currently no commercial anticoagulant that selectively and potently inhibits the medical device and not the patient.

Initial attempts to selectively anticoagulate medical devices focused on ionically- and covalently bound heparin coatings. The goal of ionically bound heparin was to slowly leach heparin from the surface to focus coagulation in the device. Any benefit this^7^ provided, however, was short-lived. The goal of covalently bound heparin was to tether the heparin at the surface using long-chain spacer polymers, enabling long-term, continued inactivation of FXa and thrombin only within the device. These coatings were able to reduce protein adsorption and clot formation only over short periods (<6 h).^15,16^ However, further study demonstrated that the bound heparin had a marked decrease in its ability to bind ATIII and inhibit FXa and thrombin and that the key advantage of these coatings was actually their ability to decrease adsorption of proteins that initiate and accelerate coagulation.^17^

As a result, research began to focus on inhibiting protein adsorption on the surfaces of these devices via surface grafting of albumin, polyethylene glycol (PEG), and zwitterionic polymers, including 2-Methacryloyloxyethyl phosphorylcholine (MPC), polysulfobetaine (PSB), and polycarboxybetaine (PCB). Of these coatings, zwitterionic materials quickly proved to be the most effective at limiting protein adsorption from blood due to their ultrahydrophilic nature. Research over the past 15 years has proven that these materials are effective at limiting nonspecific protein adsorption from blood, and current work focuses on demonstrating and optimizing that effectiveness from periods of days to weeks to months in a variety of medical devices.

## Protein Adsorption and Blood Activation in Medical Devices

**Figure 1:**
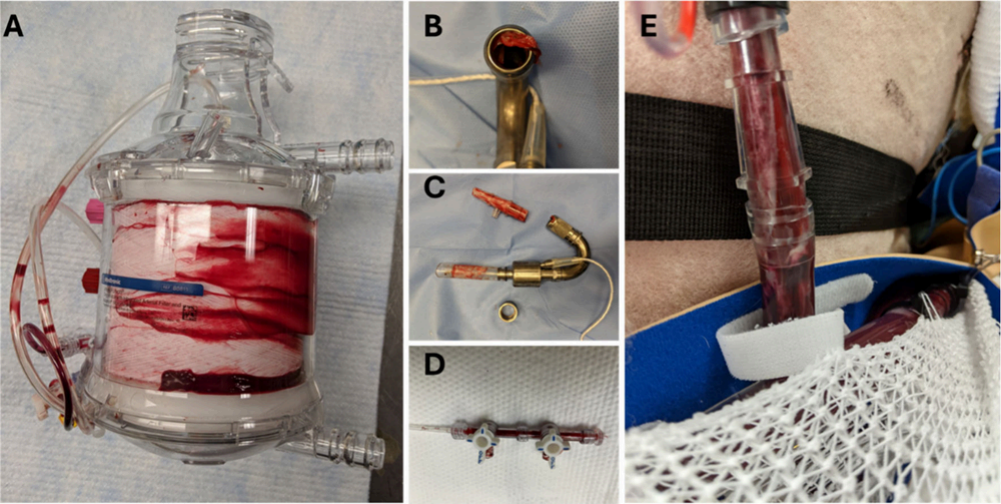
Clot formation in blood bearing medical devices: A) the large internal surface area of an oxygenator and its tubing fouled with blood clot; B) and C) clots on blood pumps; and D) and E) stopcocks and connectors occluded by blood clot.

Most blood bearing medical devices are constructed from hydrophobic polymers such as polycarbonate, polyurethane, polypropylene, polydimethylsiloxane, and polytetrafluoroethylene. A smaller subset, primarily ventricular assist devices, are constructed from titanium and stainless steel. Each of these materials rapidly adsorbs proteins from blood. While numerous proteins are adsorbed, a few have an outsized impact on device function due to their ability to activate coagulation and inflammation. Adsorption of FXII initiates a conformational change and activation to Factor XIIa.^18−20^ Factor XIIa (FXIIa) then initiates the intrinsic branch of the coagulation cascade, leading to activation of the common coagulation cascade and formation of solid clot (Figure 1). Surface adsorption of fibrinogen also induces a conformational change,^21^ enabling platelets to bind to fibrinogen via their Gp IIb/IIIa receptors, which activate them to release their numerous procoagulants and further accelerate the coagulation process.

Protein adsorption also can lead to activation of leukocytes and inflammation. Factor XII adsorption activates the intrinsic branch of the coagulation cascade, forming FXIIa and kallikrein, and complement adsorption leads to the formation of complement fragments C3a, C4a, and C5a.^22−25^ These factors, in turn, activate neutrophils, monocytes, basophils, and mast cells. In high surface area systems, such as cardiopulmonary bypass, extracorporeal membrane oxygenation, and kidney dialysis, this can cause a systemic inflammatory response that is broadly damaging to major organs.

Coagulation and inflammation, and their negative consequences, could be markedly reduced in blood bearing medical devices if protein adsorption could be reduced. The focus of surface coating research over the last 30 years has, therefore, focused on surface grafting of long chain molecules that form polymer networks or dense, brush-like structures to resist protein adsorption. These coatings include polyethylene glycol (PEG), oligoethylene glycol (OEG), Poly 2-Methacryloyloxyethyl phosphorylcholine (pMPC), polysulfobetaine methacrylate (pSBMA), and polycarboxybetaine methacrylate (pCBMA). While PEG and OEG proved effective at generating mild decreases in protein adsorption, they are less hydrophilic than the zwitterionic coatings, break down relatively quickly, and elicit an immune response.^26,27^ This makes them poorly suited for use in long-term blood-bearing medical device applications, where coatings are most needed. Thus, this review will focus on zwitterionic surface coatings due to their greater hydrophilicity, resistance to protein adsorption, and longevity.

## Zwitterionic Coating Structure and Function

**Figure 2:**
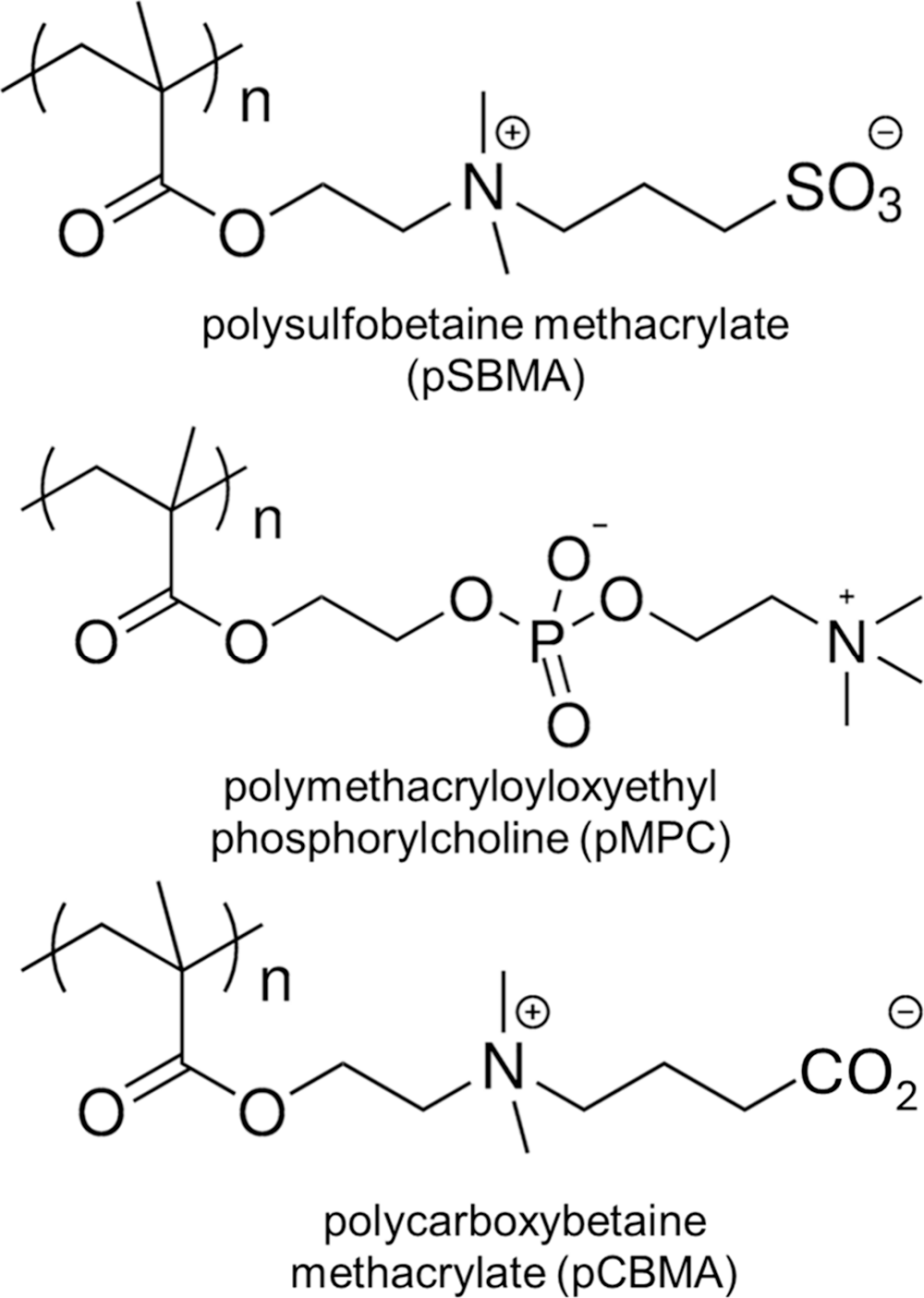
Select zwitterion side groups used as hydrophilic coatings.

The structures of pSBMA, pMPC, and pCBMA are shown in Figure 2. Each polymer has a set of common structural and chemical characteristics: (1) each features a backbone of repeating polymer segments that is anchored to the substrate through a variety of linkers, (2) each segment has a polar, zwitterionic side group, and (3) the separation between the side groups tends to be short. Zwitterions are the key to these coatings’ antifouling and anticoagulant properties. The positively charged amine groups, paired with the negatively charged carboxyl, sulfonate, and phosphate groups, define the polar, zwitterionic nature of the side groups. This polarity promotes hydration through electrostatic interaction with water. To generate effective antifouling, the substrate is coated with high packing densities of the zwitterionic polymer. If sufficiently dense, when the medical device is primed with an aqueous solution (saline, lactated Ringer’s solution, etc.), the water occupies the spaces between the zwitterionic groups. This creates a dense hydration layer over the device’s surfaces that repulses proteins. When the coated surface contacts blood, proteins such as FXIIa and fibrinogen would thus need to displace the water to reach the hydrophobic surface, adsorb, and initiate their procoagulant functions. Therefore, to be an effective coating, the substrate must have sufficient packing density to generate a tightly bound hydration layer that effectively serves as an energetic barrier that proteins need to overcome.

The ability of any of these coatings to resist fouling is dependent on their chain lengths. Longer chain lengths promote a thicker hydration layer, enhancing the material’s resistance to nonspecific protein adsorption while shorter chain lengths provide a thinner hydration layer, which may be less effective in preventing fouling. However, graft density can be higher with shorter chains due to reduced steric hindrance when compared to longer chains. Further, hemodynamic stresses may impact longer chain grafts more negatively as they can get entangled while shorter chains will exhibit less mechanical instability. Ultimately, the optimal chain length of zwitterionic grafts is one that favors thicker, denser hydration layers with excellent mechanical stability.^28−31^

## Coating Methods

The most commonly coated medical device materials are polydimethylsiloxane (PDMS), polyurethane (PU), polycarbonate, polymethylpentene (PMP), titanium, and stainless steel. As these surfaces are generally inert to direct modification, they require some preprocessing before the coating process can be initiated. For polymeric materials, the preprocessing may involve plasma exposure, etching, etc. to cleave the bonds in the polymer chains that are at the surface for (1) initiator attachment for the purpose of graft-from coating or (2) functional group attachment for the purpose of graft-to coating. If the substrate is metallic, then other mechanisms for functionalizing the metal surface for subsequent grafting can be applied after surface cleaning. Surface grafting, dip-coating, and spray coating are typical methods employed for polymeric surfaces while vapor deposition, electrophoretic deposition, surface grafting, sol–gel coating, and dip-coating are generally applied to metals. A summary of all the methods is included in Table 1, but chemical vapor deposition, self-assembled monolayers (SAM), dip coating, and surface grafting have been particularly useful in coating blood bearing devices composed of either polymeric or metallic surfaces.

**Table 1:** Blood Bearing Device Coating Methods

Coating Methods	Brief Description
Grafting (Graft to and Graft from)	Grafting to Involves attaching preformed hydrophilic polymers to the device surface.
	Grafting from Initiates polymerization directly from the device surface, growing polymer chains in situ.
Self-Assembled Monolayers	Hydrophilic molecules are adsorbed onto the device surface, forming a monolayer through spontaneous self-assembly driven by chemical affinity.
Photopolymerization	A light-sensitive hydrophilic monomer is polymerized onto the device surface using UV or visible light, creating a cross-linked hydrophilic coating.
Sol–Gel Coating	Involves the transition of a solution system from a liquid “sol” into a solid “gel” phase, resulting in a uniform hydrophilic coating after drying and curing.
Physical Vapor Deposition	Sputtering: This technique involves ejecting material from a target to coat a substrate, creating thin, uniform coatings.
	Evaporation: Material is vaporized and then condensed onto the medical device surface, forming a coating.
Chemical Vapor Deposition	This process involves reacting gaseous precursors at elevated temperatures to form a solid material on the device surface, resulting in a durable and uniform coating.
Dip Coating	The device is immersed in a hydrophilic polymer solution and then withdrawn at a controlled rate, allowing a thin film to form on the surface. The coating is then dried and cured.
Spray Coating	A hydrophilic material is sprayed onto the device surface, creating a uniform coating.
Spin Coating	A small amount of coating solution is applied to the center of the device, which is then spun at high speed to spread the solution uniformly by centrifugal force.
Layer-by-Layer (LbL) Assembly	This technique involves sequentially dipping the device in oppositely charged polyelectrolyte solutions, building up multilayered hydrophilic coatings.
Plasma Polymerization	Plasma is used to polymerize monomers directly onto the device surface, forming thin hydrophilic coatings with good adhesion and uniformity.
Electrophoretic Deposition	Hydrophilic particles suspended in a liquid medium are deposited onto the device surface under the influence of an electric field.
Self-Segregating PDMS	PMDS devices are constructed from a dilute mixture of PDMS-zwitterion copolymers and PDMS, and the copolymers self-segregate to the aqueous surface.

The chemical vapor deposition (CVD) coating process deposits gaseous precursors of the coating material onto the substrate (e.g., cardiovascular stents with titanium nitride coatings), typically at high temperatures. The substrate is exposed to one or more volatile precursors (*tert*-butyl peroxide, a chain growth monomer, silane or tetraethylorthosilicate) that react and decompose on the surface, forming a solid, thin film for further functionalization. Substrates like poly(styrene) (PS), poly(ethylene terephlate) (PET), or silicon treated with a phenyl silane coupling agent contains an aromatic group, and the precursor oxidant FeCl_3_ can be utilized to create surface radicals from which a step growth polymerization of a conducting polymer brush can proceed. It can deposit uniform, conformal coatings even on complex shapes, and thin, dense films with excellent adhesion are achievable. However, due to the high temperature requirement, temperature sensitive materials are not suitable. Additionally, CVD requires expensive equipment.^32−35^

The dip-coating process involves immersing a device (e.g., catheters) into a coating solution and then withdrawing it at a controlled speed to deposit a thin film of the coating material. The thickness depends on the withdrawal speed and solution properties. This method is simple and cost-effective, scalable for large batches, and good for applying uniform thin films on regular surfaces. However, it is limited to simpler geometries, coating thickness can vary with irregular surfaces, and postprocessing is often needed for curing or drying.^36,37^

A SAM forms when molecules spontaneously organize themselves into a single, ordered layer on a surface, typically by chemisorption.^38,39^ It is relatively easy to prepare and forms a well-defined coating with specific functional groups. For example, oxide substrates (e.g., metal stents or ventricular assist devices) can be “SAMed” with silanes and electrode sensors with thiols.^40^ SBMA can then be covalently attached to the silane “SAMed” surface following the exposure of the surface to bromoisobutyryl bromide solution at room temperature.

Surface grafting involves attaching molecules, including large polymers, to the surface of a device. This can be done through chemical reactions (e.g., grafting-from or grafting-to) or by initiators. In the graft-from approach, polymer chains are grown directly from the surface itself. This is achieved by first attaching initiators or active sites on the surface, and then using these initiators to start polymerization reactions in which monomers are added to these active sites, and polymer chains are grown outward from the surface. In the graft-to process, preformed chains are attached to substrates. Plasma treatment is often a precursor to surface coating and further modification steps for stabilization and functionalization are also applied after coating. Artificial lungs,^7,41−44^ vascular grafts,^45−51^ and catheters^52−55^ have been modified using this method.

Recently, researchers have investigated generating a zwitterionic coating on PDMS via the bulk polymer itself.^56,57^ In brief, small amounts (<2 wt %) of PDMS-zwitterion copolymers are incorporated within the PDMS during manufacture. The copolymers then spontaneously self-segregate to surfaces in contact with aqueous solutions, thus effectively coating the outer PDMS surface with a zwitterionic layer to resist nonspecific adsorption. The potential benefit of this approach is eliminating the coating step when manufacturing PDMS devices, such as PDMS microfluidics.

The optimal choice for a coating method is likely dependent on the blood-bearing medical device material; whether the surface requires maintenance of diffusive or convective mass transfer; and whether the surface is a part of a complex, three-dimensional medical device. As discussed previously, CVD is more appropriate for metals, due to its high temperatures. Devices that facilitate mass transport may require dip-coating or surface grafting, depending on the geometric complexity of the blood contacting surfaces. Mass transport is a critical factor for the function of artificial lungs, dialyzers, and electrode sensors. Artificial lungs must maintain efficient gas diffusion; dialyzers must maintain effective convective and/or diffusive fluid and waste removal during hemodialysis; and glucose sensors with implantable electrodes must maintain effective glucose diffusion. As such, coating methods that apply thin films (10–50 nm) with high permeability are likely required. Dip-coating works better on sensors due to their simplicity while the wash-through coating methods are optimal for coating whole, internal flow devices like artificial lungs and dialyzers. In contrast, ventricular assist devices, catheters, and vascular grafts do not have mass transfer or high surface permeability requirements and can, therefore, be modified with thicker coatings to achieve greater durability, resistance to fouling, and if needed, drug release.

**Figure 3:**
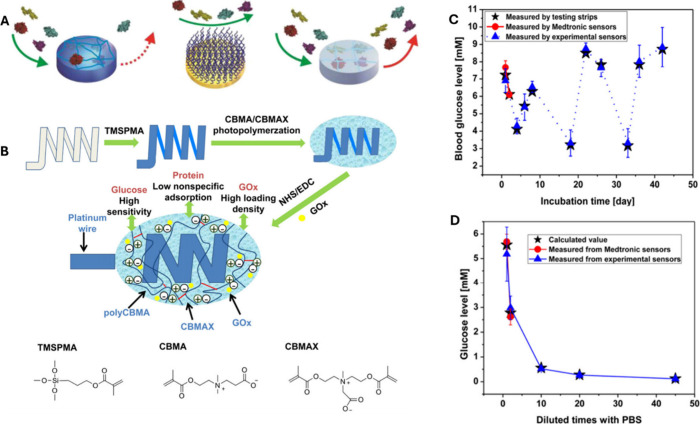
A) Schematic illustration of (left to right) a low-fouling conventional hydrogel showing protein entrapment and adhesion; a polymer brush surface coating, which highly resists nonspecific protein adsorption; and a low-fouling, lightly cross-linked hydrogel, which allows the free movement of proteins in and out of the hydrogel matrix without nonspecific protein adsorption. B) Preparation process of a glucose sensor coated with a pCBMA hydrogel lightly cross-linked with a CBMA cross-linker (CBMAX) and loaded with covalently immobilized GOx, leading to high GOx loading density, high glucose detection sensitivity, and very low nonspecific protein adsorption. C) Comparison of the experimental glucose sensor coated with 0.1% polyCBMA hydrogel with the Medtronic sensor in whole blood taken from rats, showing the blood glucose level is accurately measured by the uncoated, commercial sensor for less than 2 days and by the coated sensor for 42 days. D) Comparison of sensor sensitivity: blood glucose level is plotted as a function of PBS dilution. TMSPMA: 3-(trimethoxysilyl)propyl methacrylate. NHS/EDC: *N*-hydroxysuccinimide/1-ethyl-3-(3-dimethylaminopropyl)carbodiimide. GOx: glucose oxidase. Adapted with permission from ref (58). Copyright 2012 Biomaterials.

**Figure 4:**
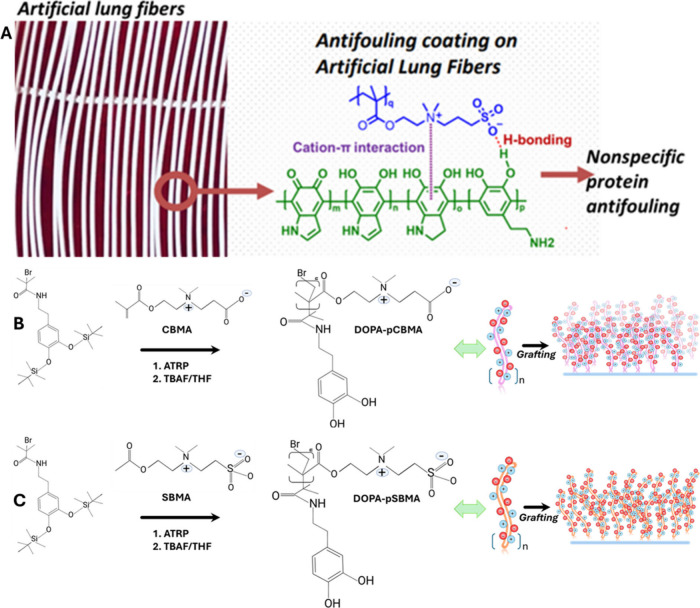
Achieving surface-focused anticoagulation in oxygenators using surface grafting with antifouling zwitterions conjugated with dopamine adhesive linkers. A) Coating of oxygenator fibers with dopamine-zwitterion (DOPA-zwitter) grafts using a wash through approach. B) Dopamine-polycarboxybetaine methacrylate (DOPA-pCBMA) and C) Dopamine-polysulfobetaine methacrylate (DOPA-pSBMA) were synthesized via solution atomic transfer radical polymerization. The reaction sequence starts with the reaction of dopamine (DOPA) linker with 2-bromoisobutyl bromide (Br-i-Bu-Br) initiator to form DOPA-Br and then the reaction of DOPA-Br with polycarboxybetaine methacrylate (CBMA) or polysulfobetaine methacrylate (SBMA) for form DOPA-pCBMA or DOPA-pSBMA. The substrate’s surface modification with DOPA-pCB polymer via pseudo one step “graft-to” coating approach was then applied. 1:6 free DOPA to DOPA-pCB were blended into a 2.5 mg/mL in tris(hydroxymethyl)aminomethane (TRIS) buffer (pH = 8.5) and was used to bathe substrates for 6 h.

As an example, the Jiang group developed a lightly cross-linked polycarboxybetaine hydrogel coating (Figure 3) to a glucose sensor using dip coating. This coating allowed glucose to diffuse through the hydrogel matrix, enabling accurate glucose measurement, while the coating inhibited sensor fouling to maintain accurate measurement while in whole blood contact for 42 days.^58^ Similar materials have since been applied to glucose sensors to improve their signal-to-noise ratio.^59^ Similarly, the Jiang and Cook groups developed a wash-through, graft-from coating method^7,41,43,44^ for artificial lungs due to the geometric complexity of the device as well as the need to maintain efficient gas exchange. This method used adhesive polydopamine (pDOPA) to graft pCBMA and pSBMA to the entire interior of artificial lungs (Figure 4). This coating process maintains a thin coating (<50 nm) to maintain gas transfer efficiency, is capable of coating multiple types of hydrophobic polymers, and reduces protein adsorption by greater than 90%.^41,43^

## Performance of Zwitterionic Coatings on Flat Surfaces

Development of zwitterionic surface coatings has typically progressed from initial testing under simplified, idealized conditions to more complex, realistic conditions. Accordingly, initial evaluation of nonspecific protein adsorption on pSB- and pCB- SAMs occurred on gold and glass surfaces under static incubation from single protein solutions. In this setting, protein adsorption was (<3 ng/cm^2^) as measured by surface plasmon resonance sensors.^26−28^ Similar results were then observed when these coatings were exposed to 100% blood plasma. When pSB was grafted to the NH_2_-terminated substrate at pH 8.5 by the SAM approach, the coating surface had undetectable adsorption from single-protein solutions (<1 mg mL^–1^ fibrinogen or lysozyme in PBS) and effectively resisted protein adsorption from 100% plasma and serum, allowing only 8 ± 7 and 22.5 ± 7.5 ng/cm^2^, respectively. Thereafter, graft-from DOPA-pCBMA was applied to PDMS membranes, which were then tested under the more realistic and challenging setting of flowing, citrated, platelet rich plasma. In this setting the DOPA-pCBMA coating reduced platelet binding by 77%.^41^

A summary of the antifouling levels (Table 2) exhibited by different types of zwitterionic coatings on model surfaces can be considered as somewhat of a benchmark of surface modification of blood bearing medical devices for antifouling. Carboxybetaine, sulfobetaine, and phosphorylcholine coatings can reduce protein adsorption to levels of less than 5, 10, and 10 ng/cm^2^, respectively. However, the test setting of these benchmarks does not reflect that of the complex test environments of coated blood-bearing devices which include different substrate materials, complex 3D structures and fluid mechanics, and whole blood interaction with the surface.^30−34^

**Table 2:** Antifouling Activity Levels of Zwitterionic Polymer Coating Type^29,60−63^

Coating Type	Protein Adsorption (ng/cm^2^) 100% blood plasma
Carboxybetaine	<5
Sulfobetaine	<10
Phosphorylcholine	<10

Due to their antiadsorptive function, zwitterionic coatings are also resistant to bacterial adhesion and are expected to be able to reduce inflammation. *P. aeruginosa* bacterial density on graft-to pCB coated glass surfaces was less than 7% of that on the glass surface at 15 h of incubation. Additionally, grafting of DOPA-pSBMA polymers to glass led to a 99.6% reduction in bacterial adhesion after 2 days when compared to an uncoated control.^64^ Lastly, zwitterionic coatings should, in theory, be able to reduce systemic inflammation by reducing adsorption and activation of the contact system of the coagulation cascade and complement activation from plasma. Unfortunately, this has not yet been studied directly. Indirect evidence of the reduction of inflammation can, however, be found in studies demonstrating a reduction in capsule formation for at least 3 months after subcutaneous implantation of pCBMA and polyhydroxyethyl methacrylate hydrogel disks in pockets made on either side of the central dorsal surface^65^ in mice and reduction of the inflammatory response to neural implants after 1 week in mouse brains.^66^

## Performance of Zwitterionic Coatings on Full Devices

Evaluation of thrombosis on three-dimensional surfaces is more complex, and less commonly reported than the two-dimensional flat sheet evaluation. Flat sheets are simpler surfaces to manipulate and to achieve complete surface coverage with zwitterionic polymers. However, these types of surfaces are idealized and rarely seen in actual blood-bearing medical devices. In fact, medical device surface geometries run the gamut from a simple tubular geometry in catheters all the way to complex, packed beds of hollow fiber membranes in oxygenators and dialyzers. Thus, coating these devices is more challenging because coating solutions do not effectively wet surfaces that are deep within a packed bed or overlapping with other surfaces. Furthermore, the blood flow velocity and patterns vary widely in these devices, ranging from low-shear, creeping flow in mass exchangers to high-shear, potentially turbulent flow in blood pumps.

### Catheters

Catheters such as peripheral venous and central venous lines and Swan-Ganz catheters are tubes with one or more lumens intended for blood sampling and pressure measurements. The most common materials used in this application include polyvinyl chloride, silicone, and polyurethane.^67^ Clinically, catheters are first inserted peripherally into patient’s vasculature, and for longer catheters like central venous lines and Swan-Ganz, their tips are advanced centrally closer toward the heart. Central venous catheters are commonly used during critical care monitoring and cancer treatments; however, their prolonged duration of use is associated with increased risks of thrombosis and bloodstream infection. In the United States, there are over 30,000 central line associated bloodstream infections each year in intensive care and acute care facilities.^68^ These occur in spite of antibacterial and antiseptic materials that may be coated or impregnated on catheter surfaces, such as silver, minocycline, rifampicin, and chlorohexidine. While these coated catheters have shown to reduce the risks of bloodstream infections relative to uncoated in clinical settings,^69^ these do not directly or indirectly inhibit thrombosis. Catheter thrombosis is less commonly reported but can occur and propagate to more serious clot complications like deep vein thrombosis.^70,71^ Zwitterionic surface coatings may therefore address both of these biological problems simultaneously by reducing nonspecific adsorption and adhesion of clotting factors, platelets, and microbes.

Clinically, there are no zwitterion-coated catheters on the market, but MPC and PSB coatings have been tested on central venous catheters *in vitro* and in animal models. Smith and colleagues coated polyurethane catheter surfaces with pSBMA in a graft-from approach, demonstrating a greater than 99% reduction in thrombus formation vs uncoated catheters over 4 h of *in vivo* testing and greater than 97% reduction in adhesion of a multiple bacterial strains after 24 h of incubation.^55^ Researchers have also combined zwitterionic coatings with surface nitric oxide (NO) release to enhance antimicrobial and antithrombotic effects. Nitric oxide is a signaling molecule released by the endothelium for various purposes, including short-acting platelet inhibition, and by macrophages as an antibacterial agent. This approach was first examined by the Cook and Jiang groups on flat sheets, demonstrating synergistic benefits at reducing platelet binding.^41^ Hitesh Handa and his research group then developed a multilayer catheter consisting of MPC graft-to topcoats that cover the outer and inner surfaces of the catheter, with an NO-releasing layer sandwiched in between.^54^ This combination achieved a nearly two-log reduction in bacteria colony forming units vs uncoated catheters and reduced visual thrombus formation after a 7-day jugular implantation study in rabbits. Furthermore, the combination of NO and zwitterionic coating was superior to either alone. Hou et al. from Nanyang Technological University also developed a diblock copolymer brush structure consisting of an NO donor moiety and pSBMA anchored to a polyurethane surface using a graft-from approach.^52^ Their catheter was tested in a five-day porcine model, demonstrating more than a four-log reduction in biofilm formation compared to a pristine catheter. Li et al. from Sichuan University is also developing a multimodal coating that consists of copper, lysine, and pSBMA that are grafted to the polyurethane catheter surface using DOPA.^53^ Catechol-copper coordination strengthens the coating’s adhesion to surfaces and reduces the risk of coating disintegration. Furthermore, copper catalyzes the reaction of endogenous nitric oxide donor species to release nitric oxide and is also inherently an antimicrobial metal species. Lysine further stabilizes polydopamine synthesis and also naturally has strong affinity to fibrinolytic proteins including plasminogen. The combination approach achieved over 98% reduction in clot weight in both a 1-h rabbit arteriovenous vascular shunt model and a 7-day rat jugular vein implant model.

### Vascular Grafts

Vascular grafts are artificial blood vessels commonly used to replace or to bypass the dysfunctional native vessels. Synthetic vascular grafts are most commonly made of polytetrafluoroethylene (PTFE), polyester (i.e., Dacron), and polyurethane. Occlusion due to thrombus formation is a common challenge, especially for small-bore grafts with diameters less than 6 mm. There are generally two approaches to reduce coagulation in vascular grafts: (1) promote endothelial migration and seeding or (2) antithrombotic surface coatings. In the latter category, heparin-bonded grafts better maintain patency after 1 year but not after 5 years.^72^ Thus, this presents an opportunity for developing novel polymer coatings to further improve long-term vascular graft performance.

Zwitterionic polymer coatings, particularly MPC, have been investigated to reduce protein fouling and increase vascular graft longevity. Yoneyama et al. evaluated a random copolymerized blend between polyurethane and MPC as a coating for 2–3 mm diameter Dacron graft. In a rabbit carotid artery end-to-end anastomosis model, they demonstrated that a 10% MPC blend maintained patency in three of the four rabbits for 8 weeks without anticoagulation.^48,49^ A research team led by Dr. William Wagner and David Vorp synthesized their own biodegradable, poly(ester urethane) urea graft with electrospinning, treated the luminal surface with ammonia plasma to functionalize it with amine groups, and then attached MPC polymers.^45,51^ Coated and uncoated vascular grafts were assessed in a rat abdominal aorta interposition graft placement model with a daily antiplatelet regimen. At the 8-week mark, the coated group maintained patency in 11 out of 12 rats, versus only 6 out of 15 in the uncoated group. One of the MPC-coated grafts maintained patency for 24 weeks.

Carboxybetaine and sulfobetaine polymers have also been studied for vascular graft applications, though not as extensively as phosphorylcholine. Drs. Sang-Ho Ye and William Wagner also developed a biodegradable, polyester sulfobetaine urethane urea graft material.^47^ During *in vitro*, whole blood, incubation studies, the SB coating reduced platelet binding by 95% following 2 h of blood contact. Wang et al. synthesized biodegradable vascular grafts made of a keratin-based hydrogen disulfide donor, polycaprolactone, and a surface coating of PCB using polydopamine.^46^ This graft was ultimately tested in a 1-month rat abdominal aorta implantation model. Their graft maintained its patency throughout the period, while displaying signs of vascular remodeling and controlled degradation of the original graft. Unfortunately, the group only reported data from only a single animal. Thus, while these results are positive, more long-term *in vivo* data are needed to evaluate the effectiveness of PSB and PCB for vascular grafts.

### Artificial Lungs

Artificial lungs, commonly called “oxygenators”, contain dense bundles of layered, hollow fiber membranes with large, 1.3–1.8 m^2^, surface areas for adult applications. These semipermeable membranes are typically composed of polypropylene (PP) or polymethylpentene (PMP) with occasional devices with thin (≈1–2 μm) PDMS surface coatings. In addition to these surfaces, artificial lungs typically feature polycarbonate housings and polyurethane (PU) potting used to separate gas manifolds from blood passages. Blood flows externally to the hollow fibers at flow rates of 3–7 L/min and blood flow velocities of 50–135 cm/min for periods of several days to weeks. Due to the densely packed, high surface area fiber bundle, long periods of blood exposure, and relatively low blood flow velocity, artificial lungs are among the most procoagulant and prone to failure of all commercial blood-bearing medical devices. The median time of first artificial lung failure in most clinical studies is 8–9 days, with many devices developing occlusive clot formation that causes device dysfunction or failure within a few weeks.^73−81^ Furthermore, the densely packed fiber bundles have overlapping hollow fibers and weaving fibers that result in numerous small areas that are challenging to coat effectively and feature static blood flow and poor washout of activated procoagulants. As a result, artificial lungs are the most challenging testbed for zwitterionic surface coatings.

Phosphorylcholine is the only zwitterionic polymer that is commercially available as an oxygenator coating. Phosphorylcholine coatings are marketed as Phisio coatings in LivaNova INSPIRE and Eurosets AMG oxygenators. Clinical reports on these oxygenators are heavily focused on patient outcomes and systemic inflammation following cardiopulmonary bypass surgeries. In these procedures, oxygenators are used for less than 6 h with a high degree of systemic anticoagulation, often without an uncoated control.^82−84^ As such, the coating’s ability to resist clot formation is not commonly documented. Clinical reports of phosphorylcholine coating performance during longer-duration ECMO also do not report on their antithrombotic performance.^85^ Thus, there is no evidence which proves that the commercial, Phisio, phosphorylcholine coating provides substantial surface-based anticoagulation in the challenging, long-term ECMO setting.

**Figure 5:**
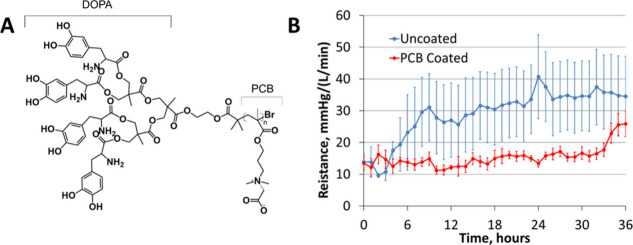
Artificial lung coating via pCBMA using a 4-DOPA attachment method (A) and resulting in vivo, artificial lung blood flow resistance vs time (B) demonstrating reduced clot formation leading to lower blood flow resistance over 36 h. Adapted with permission from ref (44). Copyright 2019 ACTA Biomaterialia.

This lack of evidence has motivated the development of new zwitterionic surface coatings that can resist thrombus formation on artificial lungs. The first such coating was developed through collaboration from the laboratories of Dr. Shaoyi Jiang and the authors of this review article. That collaboration focused on exploring a facile coating approach for surface modification of the artificial lung.^41,43^ A method was desired to provide a wash-through coating method that does not disrupt the normal artificial lung manufacturing process and is capable of coating the different hydrophobic polymer surfaces within an oxygenator, including PP, PMP, polycarbonate, and PU. Thus, Sundaram et al. first developed a flow-through, graft-from approach using DOPA to anchor pCBMA to the surfaces.^43^ Following successful studies on flat sheets^41^ (see also Performance of Zwitterionic Coatings on Flat Surfaces), these coatings were then tested on full devices. Ukita et al. then evaluated three types of grafting approaches in miniaturized artificial lungs (mini-lungs): graft-to using either (a) four DOPA or (b) random block copolymerization with hydrophobic moieties and (c) graft-from approach using atom transfer radical polymerase (ATRP).^44^ Each coating was applied in mini-lungs with PDMS-coated polypropylene hollow fiber membranes. These three types of coated devices were then placed in parallel with an uncoated control and simultaneously tested in a sheep model of veno–venous ECMO using no systemic anticoagulation for 36 h. In this highly procoagulant setting, uncoated controls began to clot and fail after 7 h, with 60% failed by hour 36. In contrast, the DOPA-pCBMA coated devices began to fail at hour 35, and only 20% had failed by hour 36 (Figure 5). There was little to no benefit in the ATRP and block copolymer groups. A follow-up rabbit ECMO experiment then evaluated the circuit coated tip-to-tip with the DOPA-pCBMA approach, and the coating reduced clot formation by 59% compared to the uncoated control.^44^ The team’s subsequent investigation reported on combining the DOPA-pCBMA coating with a bicyclic peptide Factor XIIa (FXIIa) inhibitor during short-term (1 h) rabbit ECMO.^7^ The pCBMA coating alone reduced clot formation by 75% vs uncoated controls using heparin anticoagulation, while the combination of pCBMA coating with FXIIa inhibition reduced clot formation by 94%. Furthermore, the combination of pCBMA coating plus FXIIa inhibition preserved normal tissue coagulation. Lastly, Amoako et al. recently examined coating artificial lungs with commercially available pSBMA.^86^ Resistance to protein adsorption by this coating was less effective than other, similar studies (45% of the control), but the coating was similarly effective at reducing platelet adhesion (16% of the control) following 90 min of incubation with platelet-rich plasma.

Dr. William Wagner, Sang Ho Ye, and William Federspiel have developed zwitterionic sulfobetaine block copolymer coatings for artificial lung applications.^87,88^ Initially, their effort focused solely on the hollow fiber membranes, and used fibers that were initially aminated or hydroxylated to enable a graft-from polymerization approach. These coatings demonstrated an 80–95% reduction in platelet deposition after 3 h of whole blood exposure. Over time, this team has transitioned to establishing a more universal coating approach that can provide surface coverage across multiple types of hydrophobic polymers that exist within an ECMO circuit.^87^ In their most recent work, they presented their pSB coating copolymerized with epoxy and siloxane, which showed versatility in the types of surfaces that can be coated, aqueous solubility, and the minimal need for plasma oxygen surface pretreatment.^89^ The team also demonstrated that when the coating was incorporated onto a full-scale artificial lung made with a PMP fiber bundle, oxygen and carbon dioxide transfer rates were not significantly affected by the presence of coating, and platelet deposition was reduced by approximately 85% after 2 h of whole blood contact.

The lab of Yong-Kuan Gong has developed a cross-linkable, phosphorylcholine coating for artificial lungs. Their original coating was developed from a copolymerization scheme between zwitterionic phosphorylcholine, hydrophobic butyl methacrylate, and cross-linkable trimethoxysilyl side chains, such that the coating is grafted using hydrophobic interactions and further anchored to surfaces by cross-linking.^90,91^ Their polymer coating has been applied on bare polypropylene hollow fiber membrane and tested for 2 h in their canine model of ECMO. In this animal study, the coating showed significantly better preservation of fibrinogen and platelet counts in blood and reduced platelet activation and thrombus burden on fiber surfaces when compared to the uncoated controls.^90^ In more recent studies, this group has been incorporating heparin molecules into their phosphorylcholine coating for added anticoagulation effect, and using it to coat not only artificial lung membrane surfaces but also pumps, tubing, and connectors. They have also used parallel ECMO circuits, similar to Ukita et al.,^44^ in a pig model to confirm the efficacy of the coating in a clinical oxygenator for a period of 16 h.^92^ At the same time, this group has primarily used bare microporous polypropylene for the artificial lung fiber bundle in their studies, which is contraindicated for extended ECMO use due to the risk of plasma leakage through its micropores.^93^

In sum, zwitterionic coatings on artificial lungs have demonstrated their ability to reduce protein adsorption, platelet binding, and clot formation over periods of up to 36 h. This is despite these devices being the most challenging testbed of all blood bearing medical devices. However, further work is needed to examine their function vs uncoated controls over more typical ECMO periods of one to 3 weeks.

### Artificial Kidneys

Dialyzers (artificial kidneys), like artificial lungs, contain bundles of hollow fiber membranes with surface areas on the order of 1–2 m^2^. These semipermeable membranes are typically composed of polysulfone (PS), poly(ether sulfone) (PES), polyester polymer alloy (PEPA), polyacrylonitrile (PAN), and poly(methyl methacrylate) (PMMA).^94^ However, the blood flow rates, blood flow patterns, and durations of blood contact are considerably different between dialyzers and artificial lungs. Dialyzers typically experience blood flows of less than 0.5 L/min and velocities of 60–180 cm/min^95^ for periods ranging from only a few hours in patients with chronic renal insufficiency to several days in patients with acute renal failure.

Additionally, blood flow within dialyzers follows a straight path parallel to the hollow fiber bundle. Anticoagulation of dialyzers is less challenging than artificial lungs due to their shorter duration of use and the flexibility to change out failed devices without significant patient complications. That said, activation of inflammation is a much greater concern during hemodialysis than during artificial lungs.^96^ The initiation of ECMO, and the initial exposure of blood to the artificial materials in the ECMO circuit leads to activation of complement and the contact system, generating numerous pro-inflammatory mediators. However, this fades after the first few days, making it a small issue over a period of support lasting a few weeks. Chronic kidney disease patients, in contrast, undergo intermittent hemodialysis three times per week for periods of only a few hours. This leads to repeated exposure of blood to artificial materials that are not yet fouled by plasma proteins and repeated activation of pro-inflammatory mediators, primarily complement.^22,24,97−99^ This, in turn, leads to endothelial dysfunction, atherosclerosis, and chronic cardiovascular disease.^97^

Published research in this space focuses on optimization of zwitterionic polymer grafting on surfaces like cellulose and polysulfone, and quantification of antifouling performance in simple media.^100−104^ These studies demonstrate reduced protein adsorption and platelet and bacterial adhesion with sustained filtration function.^100,102,104^ However, more studies are needed to evaluate contact system and complement activation in a more clinically relevant setting, including multiple hours of whole blood contact.

### Microfluidic Lungs and Kidneys

Microfluidic technology is gaining attention for achieving small-scale, compact organ support devices. Theoretically, these devices can achieve mass transfer profiles that are more efficient in space than the conventional artificial organ technology by minimizing the diffusion lengths. But due to the narrow cross-sectional area, the blood channels are prone to rapid occlusive thrombosis and premature failure. Surface coatings including PEG^105−108^ and heparin^109^ have been used most commonly to reduce thrombosis, while zwitterionic polymers have only been investigated by a few research teams. Dr. Joseph Potkay’s group evaluated sulfobetaine silane modification for their PDMS-based microfluidic lungs, demonstrating a 96% reduction in platelet adhesion under *in vitro* flow.^107^ Dr. William Wagner’s group also reported on their self-segregating zwitterionic group-bearing PDMS technique, formulated by mixing diallyl-terminated sulfobetaine (SB-diallyl) within a commercial PDMS base.^57^ This PDMS-SB formulation was used to fabricate microfluidic channel devices, and their blood-biocompatibility was tested by flowing blood with a low level of anticoagulation (0.625 units of heparin/mL blood). The PDMS-SB microfluidic channel was able to maintain patency for 1 h without any signs of occlusion, while the PDMS control device rapidly occluded from thrombosis. This microfabrication approach is promising as it does not require additional surface modification processes, and it may also potentially provide a more uniform surface coverage of zwitterions than a graft-to method. However, further work, particularly long-term in vivo testing, is still needed. Nevertheless, recent investigations by Borenstein’s group and their collaborators showed 24-h maintenance of their microfluidic lung device at a blood flow rate of 750 mL/min in a pig extracorporeal circulation model without any surface modifications.^110,111^ While these results are encouraging, animals in these studies were given supratherapeutic heparin anticoagulation (i.e., activated clotting time above 250 s). Surface modifications of microfluidic devices will likely help reduce this heavy anticoagulation requirement. A multimodal strategy that combines zwitterionic surface coating and surface-focused anticoagulation using NO or contact system inhibitors, as discussed previously, could be promising for these devices.^7,41,54^

More recently, zwitterionic coatings have also been applied to microfluidic artificial kidneys being developed for permanent support of patients with chronic renal disease. Dr. Shuvo Roy has been developing a biohybrid artificial kidney with nanoporous silicon membranes. Dr. Roy’s group first applied PSB coatings to these membranes, demonstrating a greater than 80% reduction in albumin and fibrinogen adsorption from single protein solutions and 10% plasma^112,113^ and almost complete elimination of platelet adhesion following 2 h of whole blood exposure.^112^ Their group then went on to investigate the impact of different means of sterilization on zwitterionic coatings applied to silicon. Overall, they found that these coatings can largely maintain their coating thickness and hydrophilicity following a wide variety of sterilization methods, but that e-beam is best suited for PSB and ethylene oxide was best for PMPC.^114^ Lastly, they evaluated platelet adhesion and activation and coagulation on PSB-coated silicon for 26-days in two pigs.^112^ These studies demonstrated no clot formation using the coating, and no increase in platelet adhesion from 6 h to 26 days. However, there was increasing platelet activation over the study. Unfortunately, these studies contained no uncoated control, likely due to the great cost of these studies, leaving it unclear if the coating itself was the cause of the excellent long-term resistance to clot formation. Lastly, the team of Dr. Buddy Ratner and Dr. Jonathan Himmelfarb have applied zwitterionic surface coatings to their wearable artificial kidney, although no data on its effectiveness have yet been published.^115^

### Ventricular Assist Devices (VADs)

Unlike the other devices discussed here, artificial hearts have a relatively small surface area with a relatively high blood flow velocity. As a result, thrombosis in artificial hearts is predominantly induced by high shear, leading to platelet and von Willebrand factor activation, increased levels of fluid recirculation and stasis, and buildup of activated procoagulants and platelets. Pump thrombosis has historically been a common clinical problem, occurring as frequently as 8–15% of patients who were implanted with the HeartMate II from Abbott and Thoratec.^116,117^ Thus, the primary means of reducing thrombosis comes from careful design of device velocity and shear patterns, which was reflected in the next generation HeartMate 3 that reported thrombosis incident of only 1.1%.^118^ Nevertheless, zwitterionic surface coatings still have the potential to further reduce thrombotic complications. Of note, the lab of Dr. Wagner has worked on zwitterionic coatings on titanium surfaces and applied them to VADs. Initially, MPC was applied to the EVAHEART VAD and evaluated preclinically over periods of one to six months.^119,120^ When compared to diamond-like carbon coatings, the MPC coatings showed similar levels of biocompatibility, with potentially a small benefit to preserving platelets multiple weeks after implantation. Since that time, Dr. Wagner and Dr. Ye have covalently attached both PC and SB zwitterions to the titanium alloy, TiAl_6_V_4_, demonstrating large reductions in platelet adhesion after 2 h of whole blood exposure.^121^

## Conclusions, Current Challenges, and Future Studies

Zwitterionic MPC, PSB, and PCB coatings have been developed and tested for various blood-bearing medical device applications. This includes surface coatings of a wide variety of biomedical materials in medical-grade tubing, vascular grafts, and artificial lungs, kidneys, and hearts. Overall, the results clearly demonstrate the ability of these coatings to create large reductions in protein adsorption and platelet adhesion for several hours to a few days. The results are particularly promising for medical devices in which the predominant cause of clotting is the artificial surfaces themselves, as in artificial lungs and kidneys, rather than shear-induced platelet activation, as in ventricular assist devices. This suggests that these surfaces could play a major future role in the success of artificial lungs and kidneys intended for permanent support.^78,79,87−91^

Despite the success of these coatings, work is required to provide translational benefits in these emerging, challenging, long-term applications. While MPC coatings are now used clinically for periods of days to weeks, there is still no clear data on the performance of any of these coatings over that time frame when compared to uncoated controls. The stability of these coatings under whole blood contact has been particularly understudied and may require further coating optimization for these settings. Additionally, each medical device has its own unique geometry, blood flow velocities and shear stresses, potential mass transport through the coated surface (e.g., artificial lungs and kidneys), duration of use and manufacturing methods. Each device, therefore, may demand a slightly different coating technique, including the means of surface attachment and the coating thickness.

The relationship between device blood flow conditions and coating technique, stability, and effectiveness is also not known. This needs to be better studied so that the ideal coating approach can be applied for the specific device application. Multiple means of coating failure are possible, including mechanical and chemical degradation. Shear can cause erosion of thin coatings,^31^ and chemical degradation is also possible, particularly during in vivo use with an active immune system capable of molecular degradation. As a result, additional means of stabilizing surface coatings may be necessary for applications lasting multiple days to weeks, including cross-linking, thicker coating layers, modified long-chain backbone chemistry, and coatings with the potential for self-renewal such as self-segregating PDMS. Lastly, surface coatings that are uneven can result in multiple point failures where small regions of protein adsorption grow to larger regions of occlusive clot via platelet adhesion and clot propagation. In this case, long-term use of a device, particularly with a low level of systemic anticoagulation, can lead to occlusive clot formation even if most of the device’s surfaces remain antiadsorptive. Thus, additional work may be required to develop coating methods that coat all regions of complex medical devices equally, particularly in devices with complex geometries and flow patterns such as artificial organs.

The method of evaluating coating biocompatibility also requires standardization. The wide variation in the selection of test medium (single protein solution, plasma, or whole blood), flow condition (static incubation or flow), and duration of testing further complicates the interpretation of data between published studies. Due to cost, initial studies are likely to continue to use single protein solutions and plasma on small material samples. However, even within this, the proteins and periods of testing require greater consistency. Thereafter, *in vivo* experiments featuring whole blood exposure will likely continue to be the gold standard. These experiments are highly expensive but continue to provide the most realistic picture of coating performance by evaluating the coatings under similar biochemical and fluid mechanical settings as their clinical applications. Thus, these studies will continue to vary, based largely on the medical device being tested.

Lastly, future studies should also consider more evaluations of the relative benefits of MPC, PSB, and PCB. Each can be used to coat materials in similar fashions, but there is little information on which zwitterion provides the most benefit with the same coating methods and test setting. The amount of published data is unfortunately scant. Additional data almost certainly exists, as various groups have experimented with different zwitterions for coating the same medical device. However, due to the cost of research and our publication culture, researchers all tend to play the winner, and thus lose important information on what approaches may be good but perhaps less than ideal.

With or without this work, zwitterionic surface coatings are likely to play a growing role in the use of blood-bearing medical devices due to their effectiveness at limiting protein adsorption, platelet adhesion, and surface clot formation. This will be particularly true in the case of current medical therapies that will be applied for longer use periods, including artificial lungs, artificial kidneys, and techniques applying both, such as long-term, ex-vivo organ perfusion.
